# The Urgent Need for Transparent and Accountable Procurement of Medicine and Medical Supplies in Times of COVID-19 Pandemic

**DOI:** 10.1186/s40545-020-00256-w

**Published:** 2020-09-11

**Authors:** Jillian Clare Kohler, Tom Wright

**Affiliations:** 1 University of Toronto Leslie Dan Faculty of Pharmacy, 144 College Street, Toronto, Ontario M5S 3M2 Canada; 2 Transparency International UK, 10 Queen St Pl, London, EC4R 1BE UK

**Keywords:** Procurement, Transparency, Accountability, Corruption, COVID-19, Health policy

## Abstract

The COVID-19 pandemic has unleashed unprecedented and complex public policy issues. One that has emerged as a challenge for many countries globally is how to ensure the efficient and effective procurement of quality medical supplies. Existing corruption pressures on procurement—everything from undue influence to the outright bribery of public officials—has been amplified by the pandemic, and thus demands commensurate policy responses. We argue that transparency and accountability in procurement are essential to preventing the corruption risks that threaten the health and well-being of populations.

## Introduction

The COVID-19 pandemic has unleashed unprecedented and complex public policy issues. One that has emerged as a challenge for many countries globally is how to ensure the efficient and effective procurement of quality medical supplies. During the pandemic, governments have scrambled to secure, not only essential medicines, but personal protective equipment (PPE), ventilators, medicines, and diagnostics. A quick Google search shows that this has even been the case in countries with robust health systems, such as Canada, the  United Kingdom, and Germany [1–3]. Urgency, demand, and shortages have placed new stress on global supply chains, leaving them vulnerable to further disruption. As a result, the market-distorting effect of price gouging and the dangerous purchasing of flawed and/or falsified goods has already been observed in many countries. Notably, “procurement is an underappreciated health system function…inefficiencies… leave some of the poorest countries paying some of the highest drug prices in the world” [4]. Equally important, the procurement process is one of the greatest risks for corruption among all government functions [5].

Corruption is defined by the leading global anti-corruption organization, Transparency International, as, “the misuse of entrusted power for private gain” [6]. It is a global phenomenon that manifests in different forms and within all types of organizations [7]. Corruption can foster inequities as it skews how resources are distributed and create access barriers to public services and goods, such as essential medicines. Ideally, the good procurement of pharmaceuticals supports access to essential medicines for the population. Yet, when corruption infiltrates the pharmaceutical procurement processes, health goals can be threatened by possible consequences such as pharmaceutical shortages, inflated drug prices, and the distribution of falsified and substandard essential medicines [7]. Procurement is thus critical to efforts by governments to ensure equitable access of essential medicines for their populations, particularly during these unprecedented times of the COVID-19 pandemic.

## Corruption and procurement

Even in non-crisis times, reducing the risk of corruption in public procurement is a challenge. This holds particularly true for procurement in the health sector, given its especially lucrative and technically complex nature. If resources allocated to pharmaceutical procurement are wasted due to corruption and operational deficiencies, not only the pharmaceutical system is affected; the health system as a whole loses out on effectiveness and equity. In turn, the public may very well lose trust in governments to deliver on their commitments such as ensuring access of the population to essential medicines.

Publicly funded essential medicines are highly vulnerable to corruption if there is weak governance in the procurement system [7]. Savedoff and Grépin, accordingly, describe corrupt activities that may take place in the pharmaceutical procurement processes such as bribes for inspectors; improper bidding procedures for purchasing; diversion of the public drug supply to private practice; kickbacks for referrals; and pharmacies or drug shops selling illegal items [8]. Ware et al. also explain how bribes or kickbacks are common forms of corruption in public procurement [9]. Corruption in procurement may be individual or systemic; in China, Rose-Ackerman and Yan highlight how individuals with procurement responsibilities may engage in corrupt behavior, while the government at large may play a role by granting promotions to physicians willing to make favorable procurement decisions for firms with ties to the state [10].

Fraud in hospital procurement is also of particular risk because of the technical expertise required to purchase pharmaceuticals. Spending large sums of money on large volumes of medicines and medical equipment, hospitals have been the object of a wide range of corrupt activities, including kickbacks in procurement and the delivery of expired and substandard products [11]. There have also been several high-profile cases showing the widespread proliferation of kickbacks and bribes from pharmaceutical firms to doctors to influence decisions over prescriptions and therefore procurements. For example, this year, Novartis paid US$ 678 million to settle a fraud lawsuit, which detailed its wide-reaching kickback program, to influence doctors to prescribe specific medicines in the United States [12].

## Corruption in procurement: COVID-19

Promising breakthroughs in COVID-19 medicines research offer seemingly rare glimpses of hope in the fight against the pandemic. For example, a University of Oxford randomized clinical trial demonstrated the beneficial use of dexamethasone, a generic drug, in improving survival rates in COVID-19 patients with respiratory complications [13]. Researchers also conducted successful early clinical trials into a promising vaccine candidate, with the latest results demonstrating the vaccine’s ability to induce a strong immune response [14]. Effective procurement of these products, and others, to treat COVID-19 will depend on robust government procurement practices. Past experience illuminates how corruption in pharmaceutical procurement can distort and restrict equitable access to their life-saving properties by increasing prices, facilitating the distribution of substandard and falsified medicines, and creating shortages.

Corruption risks in procurement are even more pronounced during times of emergency. During disaster response, huge amounts of additional funding are directed to rapidly resolve a critical and complex problem, often through acquiring limited resources under large amounts of pressure. An estimated US$ 16 trillion has already been spent by governments globally on their responses to COVID-19 [15]. The massive amounts of public funding that are allocated to procurement are not to be overlooked. And what is more, existing corruption pressures on procurement—everything from undue influence and/or bribery on public officials—are further amplified by the pandemic. Efforts to rapidly procure urgent goods may require flexibility, speed, and a level of discretion that can further widen the risks of corruption. Suppliers may exploit shortages to demand grossly inflated prices. Relaxed checks and balances can result in the purchasing of sub-standard or falsified products, which undermine health security and reduce confidence in public institutions [16]. Unscrupulous politicians may use the disruption to enrich themselves or their friends.

Examples of alleged corruption during the pandemic are already bountiful in many countries. In an effort to procure N95 face masks, the United States Federal Government gave a direct award of US$ 55 million to a company that had no experience in supplying medical supplies and no recorded employees [17]. In the UK, the government directly procured 3.5 million testing kits, which later turned out to be unusable [18], while a senior procurement official for the National Health Service (NHS) in London was reported to have traded PPE for private gain [19]. In Brazil, it was reported that the Federal Government purchased masks from a supplier with ties to President Jair Bolsonaro that were 67 percent more expensive than the other supplier bids in the same procurement competition [20].

As COVID-19 threatens a global surge in substandard and falsified products [21], it is even more important to understand the complex enabling relationship corruption in procurement of medicines has with substandard and falsified medicines. If proper procedures are not in place, drug shortages can provide an opportunity for the proliferation of substandard and falsified medicines [22].

## Preventing corruption risks in procurement: The role of transparency and accountability

How can we stop the pervasive risks of corruption that threaten the health and well-being of populations? For one, we need to ensure that there is transparency in the whole of the procurement process. Transparency requires information on procurement decisions that are publicly available. This can allow prices paid for the same health products to be compared across a local, regional, or national level and curb price gouging, price manipulation, and overpayments. For example, a group of hospital trusts as part of the United Kingdom’s National Health Service (NHS) Southern Procurement Partnership collected and standardized manufacturer and price data for generic products that were being bought separately as individual trusts [23]. These products included exam gloves and disposable aprons. In comparing this data, they were able to identify priority cost-saving opportunities which amounted to 15-50% beyond current NHS best price for these products [23]. An independent report went on to say that if this data-sharing strategy was used across the NHS, the NHS could save an estimated £500m [23]. Importantly, data transparency can illuminate patterns of normal procurement behavior and identify potential outliers indicative of overpayments, collusion, or kickbacks.

We need to ensure that even in this crisis, there are accountability mechanisms in place that make governments responsive to the people they serve. This means having metrics to document what is being procured, why, where, and for how much. In this, the role of civil society is critical. An empowered civil society can ensure that problems—corruption related or otherwise—are not swept under the rug. Without this, the effectiveness of the limited resources mobilized to respond to the pandemic will be undermined. If corruption risks are not addressed, they will ultimately result in further loss of life. Public procurement is a complex and often ignored subject, but its implications for the health of citizens is significant. Failing to properly address the corruption risks in the public procurement of medicines will severely undermine the effectiveness of the global COVID-19 response.

## Data Availability

Data sharing is not applicable to this article as no datasets were generated or analyzed during the current study.

## Abbreviations

NHS: National Health Service (United Kingdom)
OECD: Organisation for Economic Co-operation and Development
PPE: Personal protective equipment

## References

[1] Leo, G. Depleted national stockpile leaves Canada reliant on China for masks, gown and other supplies during pandemic. CBC News. 2020 [cited 2020 July 14]. Available from: https://www.cbc.ca/news/canada/saskatchewan/ppe-import-china-shortage-1.5552426.

[2] Roberts, M. Coronavirus: Has the NHS got enough PPE? BBC News. 2020 [cited 2020 July 14]. Available from: https://www.bbc.com/news/health-52254745.

[3] Connolly, K. German doctors pose naked in protest at PPE shortages. The Guardian. 2020 [cited 2020 July 14]. Available from: https://www.theguardian.com/world/2020/apr/27/german-doctors-pose-naked-in-protest-at-ppe-shortages.

[4] Silverman R, Keller JM, Glassman A, Chalkidou K. Tackling the triple transition in global health procurement. Center for Global Development. 2019. Available from: https://www.cgdev.org/sites/default/files/tackling-triple-transition-global-health-procurement-brief.pdf.

[5] Organisation for Economic Cooperation and Development. Preventing corruption in public procurement. 2016. [cited 2020 July 14]. Available from: http://www.oecd.org/gov/ethics/Corruption-Public-Procurement-Brochure.pdf.

[6] Transparency International. What is corruption? Transparency International. [cited 2020 July 24]. Available from: https://www.transparency.org/en/what-is-corruption.

[7] Kohler JC, Dimancesco D. The risk of corruption in public pharmaceutical procurement: how anti-corruption, transparency and accountability measures may reduce this risk. Glob Health Action. 2020;13(sup1). 10.1080/16549716.2019.1694745. 10.1080/16549716.2019.1694745 PMC7170361 32194011

[8] Savedoff WD, Grépin K, Plummer J (2012). Health sector corruption in Ethiopia. Diagnosing corruption in Ethiopia: perceptions, realities, and the way forward for key sectors.

[9] Ware JT, Moss S, Campos JE, Noone GP, Campos JE, Pradhan S (2007). Corruption in public procurement: a perennial challenge. The many faces of corruption: tracking vulnerabilities at the sector level.

[10] Rose-Ackerman S, Tan Y. Corruption in the procurement of pharmaceuticals and medical equipment in China: the incentives facing multinationals, domestic firms and hospital officials. UCLA Pacific Basin Law J. 2014;32(1).

[11] Savedoff WD. The impact of information and accountability on hospital procurement corruption in Argentina and Bolivia. U4 Brief. 2008;8. Available from: https://www.cmi.no/publications/3027-the-impact-of-information-and-accountability-on.

[12] Neil Vigdor. It paid doctors kickbacks. Now, Novartis will pay a $678 million settlement. The New York Times. 2020 July 1 [accessed 2020 July 22]. Available from: https://www.nytimes.com/2020/07/01/business/Novartis-kickbacks-diabetes-heart-drugs.html.

[13] Dexamethasone reduces death in hospitalised patients with severe respiratory complications of COVID-19. University of Oxford News and Events. 2020 June 16 [accessed 2020 July 24]. Available from: https://www.ox.ac.uk/news/2020-06-16-dexamethasone-reduces-death-hospitalised-patients-severe-respiratory-complications.

[14] COVID-19 vaccine AZD1222 showed robust immune responses in all participants in Phase I/II trial. AstraZeneca. 2020 [accessed 2020 July 24]. Available from: https://www.astrazeneca.com/media-centre/press-releases/2020/covid-19-vaccine-azd1222-showed-robust-immune-responses-in-all-participants-in-phase-i-ii-trial.html.

[15] Cornish, L. Interactive: Who’s funding the COVID-19 response and what are the priorities? Devex. Updated 2020 July 12 [accessed 2020 July 14]. Available from: https://www.devex.com/news/interactive-who-s-funding-the-covid-19-response-and-what-are-the-priorities-96833?mkt_tok=eyJpIjoiWm1RNVpUTXpPVGN6TURJMSIsInQiOiJmb0JGNnZLVjVTR09yQmtiUWhQWkFhTko0Z3IzUXNVTW9hMXVrVDZwSWtKdytFdlV3YUlaTklibUU4bDdnRVU3MCt3cDQ0d2RnZWhNcmhkSEJXcDl0ZGFOQnhVbVcyYmZCakVBaWtYQ1owb1gxZ0RHVyt1cmtNRTZ5UmVxenlwKyJ9#disqus_thread.

[16] Heymann, David L et al. Global health security: the wider lessons from the west African Ebola virus disease epidemic. 2015. Lancet, 385, 9980, 1884 – 1901. Available from: https://www.thelancet.com/action/showPdf?pii=S0140-6736%2815%2960858-3. 10.1016/S0140-6736(15)60858-3 PMC5856330 25987157

[17] Stanley-Becker I, Butler D, Miroff N. In coronavirus scramble for N95 masks, Trump administration pays premium to third-party vendors. The Washington Post. 2020 [cited 2020 July 14]. Available from: https://www.washingtonpost.com/national/coronavirus-trump-masks-contracts-prices/2020/04/15/9c186276-7f20-11ea-8de7-9fdff6d5d83e_story.html.

[18] Savage M, Mc Kie R. Reveal cost of 3.5m unusable Covid-19 tests, health chiefs told. The Guardian. 2020 [cited 2020 July 14]. Available from: https://www.theguardian.com/world/2020/apr/11/reveal-cost-of-35m-unusable-covid-19-tests-health-chiefs-told?CMP=Share_iOSApp_Other&fbclid=IwAR121OC9zqh4tXoxu6fQ597vs6ATVcX-9DzUinXKVDxhuPMBCYHRzvbxRhc.

[19] Davies H, Goodley S. Revealed: NHS procurement official privately selling PPE amid Covid-19 outbreak. The Guardian. 2020 [cited 2020 July 14]. Available from: https://www.theguardian.com/society/2020/may/01/revealed-nhs-procurement-official-privately-selling-ppe.

[20] Potter, H. Coronavírus: Sem licitação, mandetta paga 67% mais caro para comprar máscaras de empresa de Bolsonarista. The Intercept Brasil. 2020 [cited 2020 July 14]. Available from: https://theintercept.com/2020/03/22/mandetta-mascaras-bolsonarista-coronavirus/.

[21] The Lancet. COVID-19 and risks to the supply and quality of tests, drugs, and vaccines. Updated 2020 June 17 [cited 2020 July 22]. Available from: https://www.thelancet.com/pdfs/journals/langlo/PIIS2214-109X(20)30136-4.pdf. 10.1016/S2214-109X(20)30136-4 PMC7158941 32278364

[22] Bruckner, Till. The ignored pandemic: how corruption in healthcare service delivery threatens universal health coverage. Transparency International Health Initiative. 2019 [cited 2020 July 22]. Available from: http://ti-health.org/wp-content/uploads/2019/03/IgnoredPandemic-WEB-v3.pdf.

[23] Lord Carter of Coles. Operational productivity and performance in English NHS acute hospitals: unwarranted variations. 2016 [cited 2020 July 22]. An independent report for the Department of Health. Available from: https://assets.publishing.service.gov.uk/government/uploads/system/uploads/attachment_data/file/499229/Operational_productivity_A.pdf.

